# Effects of Different Biological Therapies on S1/S2 Antibody Response to SARS-CoV-2 Vaccination in a Cohort of Patients with Inflammatory Bowel Disease

**DOI:** 10.3390/vaccines10071077

**Published:** 2022-07-04

**Authors:** Nunzia Labarile, Fabio Castellana, Annamaria Sila, Pasqua Letizia Pesole, Sergio Coletta, Margherita Curlo, Rodolfo Sardone, Gianluigi Giannelli, Mauro Mastronardi

**Affiliations:** 1 Unit of Gastroenterology, National Institute of Gastroenterology IRCCS “Saverio de Bellis”, Research Hospital, Castellana Grotte, 70013 Bari, Italy; nunzia.labarile@gmail.com; 2 Unit of Research Methodology and Data Sciences for Population Health, National Institute of Gastroenterology IRCCS “Saverio de Bellis”, Research Hospital, Castellana Grotte, 70013 Bari, Italy; fabio.castellana@irccsdebellis.it (F.C.); annamaria.sila@irccsdebellis.it (A.S.); rodolfo.sardone@irccsdebellis.it (R.S.); 3 Biobank Core Facilities, National Institute of Gastroenterology IRCCS “Saverio de Bellis”, Research Hospital, Via Turi 27, Castellana Grotte, 70013 Bari, Italy; letizia.pesole@irccsdebellis.it (P.L.P.); sergio.coletta@irccsdebellis.it (S.C.); 4 Inflammatory Bowel Disease Unit, National Institute of Gastroenterology IRCCS “Saverio de Bellis”, Research Hospital, Castellana Grotte, 70013 Bari, Italy; margherita.curlo@irccsdebellis.it (M.C.); mauro.mastronardi@irccsdebellis.it (M.M.); 5 Scientific Direction, National Institute of Gastroenterology IRCCS “Saverio de Bellis”, Research Hospital, Castellana Grotte, 70013 Bari, Italy

**Keywords:** vaccination, IBD, biological therapy, SARS-CoV-2

## Abstract

The severe acute respiratory syndrome coronavirus 2 (SARS-CoV-2) infection has affected the entire planet. The objectives of our study were to compare responses to the vaccine (Pfizer-Biontech COMIRNATY) in a population of patients with intestinal bowel syndrome undergoing different biological therapies or conventional therapy. The study recruited 390 patients who received the first vaccination dose during the dedicated vaccination campaign for inflammatory bowel disease (IBD) patients. The inclusion criteria were a diagnosis of CD or UC and complete vaccination with the Pfizer–BioNTech COVID-19 (Comirnaty) vaccine. The exclusion criteria were other significant diseases or important therapies under way or contraindications to vaccination according to the European drug surveillance recommendations. Linear rank models were run to assess the association between the different therapies and S1/S2 antibodies at three different times. The models showed that in patients with IBD receiving Vedolizumab a significant increase in mean IgG levels was observed, independently of other therapies and confounding factors (β: 57.45, 95% CI 19.62 to 19.00). This study confirmed the complete antibody response to vaccination against COVID-19 in patients with IBD undergoing biological therapy—particularly Vedolizumab treatment—but also a reduced immune response due to concomitant steroid therapy.

## 1. Introduction

After the first outbreak in December 2019, the novel coronavirus denominated SARS-CoV-2 (severe acute respiratory syndrome coronavirus 2), identified as the causative agent of a respiratory syndrome named coronavirus disease (COVID-19), rapidly turned into a pandemic [1]. In response to this pandemic, multiple effective and safe vaccines to suppress viral transmission were soon produced based on different technologies: inactivated or live attenuated vaccines, recombinant protein vaccines, vectored vaccines, and RNA and DNA vaccines [2,3]. The mRNA vaccines include mRNA-1273 produced by Moderna and BNT162b2 by Pfizer–BioNTech, which received Emergency Use Authorization (EUA) by the Food and Drug Administration (FDA) in December 2020 in people aged ≥18 years and ≥16 years, respectively, with more than 90% efficacy [4]. The goal of vaccination is to produce long-term immunity against infection through the production of pathogen-specific antibodies. Among the coronavirus’ structural proteins, the spike glycoprotein (S) and Nucleocapsid (N) proteins are the main immunogens, and have been identified as the key targets for protective antibodies against SARS-CoV-2 [5,6]. The S- protein of SARS-CoV-2 is a homotrimeric glycoprotein located on the virion surface; it plays a major role in virus entry into target cells by binding to specific entry receptors [7]. The S protein consists of two subunits: S1, which contains the Receptor Binding Domain (RBD), and S2 [8]. Studies have shown that immunization against the spike protein of SARS-CoV-2 reduces transmission rates and severe outcomes [8]. As COVID-19 has become a global issue, expert consensus also recommends vaccination programs against SARS-CoV-2 in patients with inflammatory bowel diseases (IBD) [9]. Although IBD patients are known to have an increased susceptibility to infections [10], many studies showed no difference in the incidence and mortality of COVID-19 between IBD patients and the general population [11,12]. However, the efficacy and safety issues of these vaccines in people with Ulcerative colitis (UC) and Crohn’s disease (CD) has given rise to some uncertainty, especially in relation to IBD therapies. The main treatment goal in IBD is to achieve and maintain disease remission, and over time the therapeutic armamentarium for IBD has slowly grown. In addition to conventional drugs such as 5-aminosalicylic acid (5-ASA) and immunosuppressants, refractory patients can be treated with biological therapies such as infliximab (IFX), Adalimumab, Ustekinumab, and Vedolizumab [13]. However, there is evidence that patients with IBD receiving immunosuppressive agents have a diminished response to some vaccinations (pneumococcal, influenza, hepatitis A and B, cholera vaccine) compared to control individuals [14,15,16,17,18,19]. To our knowledge, there is no reported evidence regarding the immune response following mRNA vaccination in IBD patients undergoing different biological therapies. In this single-center, clinically based observational prospective study, we aimed to assess and compare the association between different IBD biological therapies and the response to the COMINARTY vaccine, using S1/S2 antibodies as a proxy variable.

## 2. Methods

### 2.1. Study Design and Population

The proposed study was conducted in the clinical setting of the IBD Unit of the Research Hospital IRCCS “S. De Bellis” in 391 patients who were followed up periodically in the unit. Patients were recruited at the time of their first vaccination dose during the vaccination campaign dedicated to IBD patients. The only inclusion criteria were a diagnosis of CD or UC and full vaccination with the Pfizer–BioNTech COVID-19 vaccine (Comirnaty). The exclusion criteria were the presence of other significant diseases (e.g., cancer, stroke, dementia, major respiratory disease) or major therapies (e.g., chemotherapy) or contraindications to vaccination following the European Pharmacological Surveillance recommendations [15]. Patient selection was carried out with non-probabilistic convenience sampling from those who presented for vaccination. Clinical evaluation was performed only at baseline before the first vaccination (t0). Blood samples were collected on the day of the vaccination (t0), 21 days before the second dose of the vaccine (t1), and 3 weeks after the second dose (t2). The study was conducted following the Helsinki Declaration of 1975 and adhered to the “Standards for Reporting Diagnostic Accuracy Studies” (STARD) guidelines (http://www.stard-statement.org/, accessed on 8 May 2022) and the “Strengthening the Reporting of Observational Studies in Epidemiology” (STROBE) guidelines. The Institutional Review Board of the IRCCS “S. De Bellis” Institute approved the “Salus in Apulia Study”, with its measurements and data collection, in accordance with the Helsinki Declaration of 1975.

### 2.2. Clinical and Laboratory Assessment

After blood clot formation, samples were centrifuged at 3200 rpm for 10 min at room temperature and sera were stored at <80 °C. Quantitative measurement of IgG antibodies against S1/S2 SARS-CoV-2 antigens was performed using the LIAISON chemiluminescent immunoassay (Diasorin, Saluggia, Italy), according to the manufacturer’s instructions. The solid phase consisted of magnetic particles coated with biotinylated S1 and S2 antigens purified from mammalian cells; the conjugate was a monoclonal anti-human IgG antibody conjugated with an isoluminol derivative. There were two calibrators in triplicate. The detection limit for the test was 3.8 Au/mL. A low control and a qualitative control had to be considered; the low control was between n/a and 6 Au/mL while the positive control was between 15 and 45 Au/mL. The cut-off was >15 Au/mL and results between 12.0 and 15.0 Au/mL were considered borderline.

All the patients had been diagnosed at the IBD Unit of the IRCCS “S. De Bellis”. At baseline, the clinical disease activity score was assessed and recorded using the Crohn’s Disease Activity Index (CDAI) for CD, a composite endpoint which includes patient symptoms, physician assessments, and lab [15] and partial Mayo Scores (PMS) for UC, based on stool frequency, rectal bleeding, and the Physician Global Assessment [16]. The score was normalized to a scale from zero to 1 (0 = no activity to 1 = severe activity) and incorporated into a single continuous variable labeled the Disease Activity Score. Corticosteroid therapy was assessed at baseline, considered as a binary discrete variable (yes/no). The therapies considered were 9 to 3 mg Budesonide and 50 to 25 mg Prednisone in the last three days before vaccination. The patients were divided into four sub-groups according to their IBD therapies: conventional therapy (mesalazine), Anti-TNF Alpha, Ustekinumab, or Vedolizumab.

### 2.3. Statistical Analysis

As shown in Table 1, the whole sample was subdivided into 4 different groups according to their ongoing therapy: anti-TNF alpha drugs, Ustekinumab, Vedolizumab, or conventional treatment.

Shapiro’s test was applied to check the normal distribution of variables in each group. Participant characteristics were reported as median (min to max values) and IQR for continuous variables, and frequencies and percentages for categorical variables.

Due to the non-normal distribution of every variable considered, a non-parametric approach was adopted—testing differences in continuous variables with the Kruskal–Wallis sum rank test, while differences in proportions were tested with the Chi-squared test.

A multivariable, nested, rank-based estimation linear regression model was built on the S1/S2 IgG response—as the dependent variable for each vaccine administration time—to estimate the coefficient of the type of treatment, adjusted for covariates assumed to have a confounding effect. Major confounding factors such as age, gender, type of disease (CD vs. UC), and corticosteroid treatment at the time of vaccination were implemented as covariates in the adjusted model, selected from those assumed to be related to the exposure (type of treatment) and the outcome (S1/S2 IgG). The methodological approach and analyses were designed and operated by a senior epidemiologist (RS) and biostatistician (FC), using RStudio Team (2022). RStudio: Integrated Development Environment for R. version 2022.2.3.492′. R Packages: tidyverse, gmodels, kablExtra, rstatix, effsize, EpiR, Rfit, ggplot2.

### 2.4. Results

The whole sample size was 390 (N = 162 females, 41.5%) IBD patients (256 (65.6%) had CD and 134 (34.4%) had UC) with a median age of 45 years and an IQR of 22.75. No patient had vaccination failure. The median S1/S2 IgG responses over the entire sample at the three different time points were t0 3 (IQR 1.6), t1 40.7 (IcsQR 48.52), and t2 208 (IQR 156.75), respectively, depicting an important increase over time—especially after the second dose of the vaccine (t2).

A description of the whole sample according to CS treatments is provided in Table 2. Here, significance only emerged with the indices of disease activity, which were found to be higher in the CS subgroup; the latter corroborates the internal validity of the findings. According to the different therapies shown in Table 3, a relative majority of patients (N = 217, 55.6%) were undergoing anti-TNF alpha treatment, while n. 48 (12.3%) were taking Ustekinumab, n. 67 (17.2%) Vedolizumab, and n. 58 (14.9%) were undergoing no immunomodulating treatment. At the time of vaccination, 57 (14.60%) of the subjects were taking corticosteroid therapies. The IgG S1/S2 assessed in the four different therapy groups showed no difference at the three assessment times, except in the Vedolizumab group. In that group, the last assessment (T2), six months after the vaccination date, showed higher values for IgG S1/S2 than in the previous assays. The non-parametric linear models of T0 (Table 4), did not shown any significant association. The non-parametric linear models of T1, shown in Table 5, highlighted a meaningful association between Vedolizumab treatment and an increased probability of higher mean values of IgG (β: 10.71, 95% CI 5.60 to −0.26) than in the other subjects, adjusted for every other therapy, age, gender, type of disease (UC vs. CD), and corticosteroid therapy. At T2, the results depicted in Table 6 revealed a further meaningful increment in the average IgG levels in the subjects receiving Vedolizumab (β: 57.45, 95% CI 19.62 to 19.00), regardless of other therapies and confounding factors. Finally, no other covariates, including the type of disease (CD or UC), had a meaningful effect on the trajectories of IgG S1/S2 responses. In summary, vedolizumab showed a strong, meaningful association with increasing levels of antibodies independently of all the other covariates, other biological therapies, and corticosteroid therapy at the time of vaccination.

## 3. Discussion

Our study aimed to measure the association between different IBD biological therapies and the response to the Pfizer–Biontech COMIRNATY vaccine using the trajectories of S1/S2 antibodies responses at three different observation times. All the IBD patients enrolled, subdivided into four groups receiving different IBD therapies, developed an antibody response (median S1/S2 IgG response at the three different times: 3 at t0, 40.7 at t1, and 208 at t2)—demonstrating the progressive growth of the antibody titer over the three examinations. However, the major finding of this study was that, in the non-parametric models, the Vedolizumab group had significantly increased mean levels of S1/S2 IgG compared to the other groups—particularly after the full vaccination effect (21 days from the second dose of vaccine). Remarkably, this association also remained strong after adjustment for age, gender, type, and stage of IBD activity. A plausible hypothesis for the biological pathways underlying the evidence of this increased antibody response after vaccination in the vedolizumab-treated IBD subset is still far away and needs further literature to explain causal mechanisms. Additionally, our cross-sectional design did not allow for causal inferences. Therefore, any discussion on this area was not approached here. To the best of our knowledge, the current data are inconsistent and do not point in the same direction. There is, however, a body of evidence that vedolizumab reduces immunogenic potency and is safer than other biological therapies, so the use of anti-integrins in immune-mediated inflammatory diseases might have less of an impact on immunologic responses and thus on antibody loads [17,18,19].

The incidence of SARS-CoV-2 among patients with IBD appears to be comparable to that observed in the general population [20]. In December 2020, the first vaccine against COVID-19—produced by BioNTech/Pfizer—was approved, eliciting crucial protective immune responses for the prevention and mitigation of the pandemic. However, little is known about the antibody response to SARS-CoV-2 vaccination in patients with IBD undergoing therapy with immunomodulators or biologics. After other vaccinations, studies have shown that these patients have a suboptimal vaccine response, probably due to their immune system dysfunction; this is especially true in patients taking infliximab or combination immunosuppressive therapies [21,22,23,24].

The results of the study are in agreement with what was observed in the ICARUS study [25], which investigated the antibody response to the mRNA COVID-19 vaccines (Pfizer and Moderna) in 48 IBD patients compared to 43 patients without IBD [26]. Wong et al. showed robust serological responses in patients undergoing biological therapy, with 100% seroconversion and no differences in anti-Spike IgG levels between the two groups. However, in this study, unlike our data, the Vedolizumab group had lower anti-S IgG levels compared to the anti-TNF alpha therapy group. The results were different in another larger multicenter study that evaluated antibody response rates in 428 patients receiving vedolizumab and 865 receiving anti-TNF alpha [26]. As in our study population, the investigators found lower mean antibody concentrations in patients treated with IFX than vedolizumab. Of interest, in our study, the association with corticosteroid therapy at the time of vaccination determined an important decrease in the predicted IgG levels in all the groups—but this was significant only in the Vedolizumab group. As shown by Kennedy et al. [27], combination therapies with an immunomodulator further reduce immunogenicity. In conclusion, our study confirmed a complete antibody response to vaccination against COVID-19 in patients with IBD undergoing biological therapy, but a reduced immune response due to concomitant steroid therapy. However, we also confirmed the findings observed in [26] of the greatest antibody response being in patients taking Vedolizumab. Yet, it appears that in absolute terms our findings are not generalizable and consistent with other literature on the topic; in fact, as reported by Alexander and colleagues [28], in patients with IBD, the immunogenicity of COVID-19 vaccine varies with exposure to immunosuppressive drugs. These authors found an attenuated antibody response in patients taking infliximab, infliximab plus thiopurine, and tofacitinib.

The principal strength of this study is that it was conducted in a large sample of IBD patients. Moreover, it is one of the few studies that has also considered the immune response in patients taking different biological therapies, including Ustekinumab—comparing them with results in patients taking other biologics and those not taking biologics.

However, the study has some limitations: firstly, the S1/S2 IgG assay is not the gold standard for assessing immunity—particularly when considering the new SARS-CoV-2 variants and their different effects on the immune system. Additionally, our database lacks a single S1 and S2 IgG essay. Secondly, corticosteroid therapies and the clinical stage of the disease were evaluated only at the baseline examination, introducing a considerable confounding effect in the T1 and T2 examinations. Thirdly, completely different conclusions might have been drawn if antibody measurements had been made after the third dose. Moreover, we lack a comparison of our IBD cases with a subset of healthy controls.

In conclusion, in this study, patients with IBD responded to COVID-19 vaccination—as measured by S1/S2 antibody levels—leaving no doubt about the usefulness and safety of this vaccination. Remarkably, subjects undergoing Vedolizumab therapies showed an increased response compared to those undergoing other therapies, even when considering the antagonist effect of corticosteroid therapies. These results, and particularly the different antibody responses in the groups undergoing these various therapies, should be compared with similar data collected after the third dose of vaccination and/or when assessing other types of antibodies, such as neutralizing and trimeric antibodies. Further evaluation of antibody titers in different populations should be conducted over time, to reach a consensus as regards the need for early revaccination in patients with IBD.

## Figures and Tables

**Table 1:** Description of the whole sample. N: 390.

	Median (Min to Max)	IQR
Age (years)	45 (19 to 76)	22.75
Age quartiles	2 (1 to 4)	1.75
Sex		
Female	162 (41.50)	
Male	228 (58.50)	
Disease Activity score	5 (2 to 17)	4.00
Normalized Disease Activity score	0.20 (0 to 1)	0.26
Type of disease		
CD	256 (65.50)	
UC	134 (34.50)	
Type of treatment		
Conventional therapy	58 (14.90)	
Anti-TNF alpha	217 (55.60)	
Ustekinumab	48 (12.30)	
Vedolizumab	67 (17.20)	
Cortisone (yes)	57 (14.60)	
IgG SARS-CoV-2 (T0)	3 (3 to 403)	1.60
IgG SARS-CoV-2 (T1)	40.7 (3 to 2190)	48.52
IgG SARS-CoV-2 (T2)	208 (16.5 to 2350)	156.75

Abbreviations: CD: Crohn’s Disease, UC: ulcerative colitis.

**Table 2:** Description of the whole sample according to CS treatments. N: 390. All data are shown as median (IQR) for continuous variables and as n (%) for proportions.

	Without CS Treatment	With CS Treatment	
	Median (IQR)	Median (IQR)	*p* Value *
Proportions (%)	333 (85.40)	57 (14.60)	
Age (years)	46 (21)	44 (25)	0.89
Age quartiles	3 (1)	2 (3)	
Sex			
Female	137 (41.10)	25 (43.90)	0.81
Male	196 (58.90)	32 (56.10)	
Disease Activity score	5 (3)	6 (5)	<0.01
Normalized Disease Activity score	0.2 (0.2)	0.27 (0.33)	<0.01
Type of disease			
CD	224 (67.30)	32 (56.10)	0.10
UC	109 (32.70)	25 (43.90)	
Type of treatment			
Conventional therapy	45 (13.50)	13 (22.80)	0.18
Anti-TNF alpha	192 (57.70)	25 (43.90)	
Ustekinumab	40 (12.00)	8 (14.00)	
Vedolizumab	56 (16.80)	11 (19.30)	
IgG SARS-CoV-2 (T0)	3 (1.6)	3 (1.5)	0.84
IgG SARS-CoV-2 (T1)	42 (48.1)	35.3 (54.3)	0.10
IgG SARS-CoV-2 (T2)	210 (163)	185 (162)	0.12

* Wilcoxon sum rank test for independent samples for continuous variables and Chi-squared test for proportions.

**Table 3:** Description of the whole sample according to type of treatment. N: 390. All data are shown as median (min to max) and IQR for continuous variables and as n (%) for proportions.

	Conventional Therapy		Anti-TNF Alpha		Ustekinumab		Vedolizumab		*p*
Proportions (%)	58 (14.90)		217 (55.60)		48 (12.30)		67 (17.20)		
Age (years)	45 (24 to 76)	19	46 (19 to 76)	21	41 (19 to 70)	20.25	48 (22 to 75)	27.50	**0.03**
Age quartiles	2 (1 to 4)	2	3 (1 to 4)	1	2 (1 to 4)	2	3 (1 to 4)	2	0.13
Sex									
Female	30 (51.70)		86 (39.60)		19 (39.60)		27 (40.30)		0.40 ^χ^2^^
Male	28 (48.30)		131 (60.40)		29 (60.40)		40 (59.70)		
Disease Activity score	6 (2 to 13)	3	5 (2 to 15)	3	5 (2 to 15)	2	5 (2 to 17)	3	0.24
Normalized Disease Activity score	0.26 (0 to 0.73)	0.20	0.20 (0 to 0.87)	0.20	0.20 (0 to 0.86)	0.13	0.20 (0 to 1)	0.20	0.20 **
Type of disease									
CD	**30 (51.70)**		**165 (76.00)**		**37 (77.10)**		**24 (35.80)**		**<0.01** ^χ^2^^
UC	28 (48.30)		52 (24.00)		11 (22.90)		43 (64.20)		
Cortisone (yes)	13 (22.40)		25 (11.50)		8 (16.70)		11 (16.40)		0.18
IgG SARS-CoV-2 (T0)	3 (3 to 10.7)	1.77	3 (3 to 403)	1.60	3 (3 to 31.5)	1.35	3 (3 to 33.7)	1.65	0.55
IgG SARS-CoV-2 (T1)	39.9 (3 to 113)	62.08	39 (3 to 2190)	48.10	38.95 (4.5 to 981)	50.60	46 (3 to 155)	36.85	0.34
IgG SARS-CoV-2 (T2)	**211 (18.7 to 1180)**	**178.80**	**180 (39.4 to 1530)**	**135.00**	**210.5 (72.4 to 1280)**	**139**	**260 (16.5 to 2350)**	**125.50**	**<0.01**

Kruskal–Wallis sum rank test, ^χ^2^^ Chi squared test, ** Jonckheere–Terpstra test. Abbreviations: CD: Crohn’s Disease, UC: ulcerative colitis. Significance shown in bold.

**Table 4:** Rank-based estimation regression on T0 S1/S2 IgG SARS-CoV-2.

	Coef.	Std. Err.	CI 95%	Coef.	Std. Err.	CI 95%	Coef.	Std. Err.	CI 95%
	Model 1			Model 2			Model 3		
(Intercept)	3.00	0.03	2.94 to 3.06	3.00	7.04	−10.81 to 16.81	3.00	0.04	2.90 to 3.09
Anti-TNF alpha	0.00	0.03	−0.07 to 0.07	−0.01	5.36	−10.50 to 10.50	−0.01	0.03	−0.06 to 0.06
Ustekinumab	0.00	0.05	−0.09 to 0.09	0.01	6.98	−13.68 to 13.68	0.01	0.04	−0.09 to 0.09
Vedolizumab	0.00	0.04	0.08 to 0.08	−0.01	6.27	−12.30 to 12.30	−0.01	0.04	−0.0.8 to 0.08
Age quartile				−0.01	1.55	−3.04 to 3.04	−0.01	0.01	−0.02 to 0.02
Sex (male)				0.01	3.53	−6.91 to 6.91	0.01	0.02	−0.04 to 0.04
Type of disease (UC)				0.01	3.91	−7.66 to 7.66	0.01	0.02	−0.05 to 0.05
Normalized Disease activity score				−0.01	10.56	−20.70 to 20.70	−0.01	0.07	−0.14 to 0.14
Cortisone (yes)							0.01	0.03	−0.06 to 0.06

Abbreviations: CD: Crohn’s Disease, UC: ulcerative colitis.

**Table 5:** Rank-based estimation regression on T1 S1/S2 IgG SARS-CoV-2.

	Coef.	Std. Err.	CI 95%	Coef.	Std. Err.	CI 95%	Coef.	Std. Err.	CI 95%
	Model 1			Model 2			Model 3		
(Intercept)	35.75	4.43	27.06 to 44.44	55.97	6.68	42.87 to 69.07	55.93	6.72	42.76 to 69.09
Anti-TNF alpha	3.70	4.54	−5.19 to 12.59	4.68	4.84	−4.08 to 14.17	4.27	4.79	−5.11 to 13.64
Ustekinumab	6.40	5.99	−5.33 to 18.13	4.84	6.30	−7.51 to 17.20	7.67	6.23	−7.54 to 16.89
Vedolizumab	9.80	5.50	−0.98 to 20.58	10.93	5.67	−0.17 to 17.20	10.71	5.60	−0.26 to 21.69
Age quartile				−7.75	1.40	−0.17 to 22.04	−7.69	1.39	−10.38 to −4.95
Sex (male)				−1.65	3.18	−10.49 to −5.00	−1.58	3.15	−7.75 to 4.58
Type of disease (UC)				3.25	3.53	−7.89 to 4.59	3.98	3.50	−2.87 to 10.84
Normalized Disease activity score				−4.55	9.57	−3.66 to 10.17	0.57	9.74	−18.51 to −4.59
Cortisone (yes)						−23.24 to 14.14	−8.48	4.60	−17.50 to 0.53

Abbreviations: CD: Crohn’s Disease, UC: ulcerative colitis.

**Table 6:** Rank-based estimation regression on T2 S1/S2 IgG SARS-CoV-2.

	Coef.	Std. Err.	CI 95%	Coef.	Std. Err.	CI 95%	Coef.	Std. Err.	CI 95%
	Model 1			Model 2			Model 3		
(Intercept)	200.58	14.38	172.40 to 228.77	233.22	23.41	187.30 to 279.10	233.39	23.67	187.00 to 279.77
Anti-TNF alpha	−16.01	15.54	−46.46 to 14.45	−10.25	16.77	−43.10 to 22.63	−9.63	16.77	−42.50 to 23.24
Ustekinumab	14.84	20.51	−27.37 to 55.05	16.31	21.85	−26.50 to 59.13	15.18	21.83	−27.60 to 57.98
Vedolizumab	53.99	18.86	17.04 to 90.95	58.25	19.64	19.80 to 96.74	57.45	19.62	19.00 to 95.91
Age quartile				−11.72	4.86	−21.20 to −2.20	−11.94	4.85	−24.40 to −2.43
Sex (male)				−14.56	11.04	−36.20 to 7.07	−15.83	11.03	−37.40 to 5.78
Type of disease (UC)				9.53	12.23	−14.40 to 33.51	11.96	12.25	−12.10 to 35.98
Normalized Disease activity score				−21.57	33.05	−86.30 to 43.21−	−5.52	34.12	−72.40 to 61.34
Cortisone (yes)							−29.55	16.12	−61.10 to 2.05

Abbreviations: CD: Crohn’s Disease, UC: ulcerative colitis.

## Data Availability

The data presented in this study are available on request from the corresponding author. The data are not publicly available due to privacy.
